# Down-regulation of autophagy proteins is associated with higher mTOR expression in the placenta of pregnant women with preeclampsia

**DOI:** 10.1590/1414-431X2022e12283

**Published:** 2023-01-09

**Authors:** I.C. Weel, V.R. Ribeiro, M. Romão-Veiga, E.G. Fioratti, J.C. Peraçoli, M.T.S. Peraçoli

**Affiliations:** 1 Departamento de Ciências Químicas e Biológicas, Instituto de Biociências, Universidade Estadual Paulista, Botucatu, SP, Brasil; 2 Departamento de Ginecologia e Obstetrícia, Faculdade de Medicina de Botucatu, Universidade Estadual Paulista, Botucatu, SP, Brasil

**Keywords:** Autophagy, LC3, Beclin-1, mTOR, Placenta, Preeclampsia

## Abstract

Autophagy is a lysosomal degradation pathway that removes protein aggregates and damaged organelles maintaining cellular integrity. It seems to be essential for cell survival during stress, starvation, hypoxia, and consequently to the placenta implantation and development. Preeclampsia (PE) is a multisystemic disorder characterized by the onset of hypertension associated or not with proteinuria and other maternal complications. Considering that the placenta seems to play an important role in the pathogenesis of PE, the objective of the present study was to evaluate protein levels of light chain protein (LC3), beclin-1, and the mammalian target of rapamycin (mTOR) in the placenta of pregnant women with PE. Placental tissues collected from 20 women with PE and 20 normotensive (NT) pregnant women were evaluated for LC3, beclin-1, and mTOR expression by qPCR and immunohistochemistry. The mRNA for LC3 and beclin-1 were significantly lower, while mTOR gene expression was significantly higher in the placenta of pregnant women with PE than in the NT group. Placentas of PE women showed significantly decreased protein expression of LC3-II and beclin-1, whereas mTOR was significantly increased compared with the NT pregnant women. There was a negative correlation between protein expression of mTOR and LC3-II in the placental tissue of PE women. In conclusion, the results showed autophagy deficiency suggesting that failure in this degradation process may contribute to the pathogenesis of PE; however, new studies involving cross-talk between autophagy and inflammatory molecular mechanisms might help to better understand the autophagy process in this obstetric pathology.

## Introduction

The autophagic process can reduce injuries related to inflammation, infections, neoplasia, and degenerative diseases, and is involved in several pathologies (1). It is a catabolic lysosomal degradation process that maintains cellular homeostasis and promotes cell survival during stress (2). This mechanism degrades protein aggregates and damaged organelles in the cytoplasm through the formation of an autophagosome (3).

The autophagosome is composed of a double lipid bilayer membrane-bound structure that merges with lysosomes forming the autolysosome, which is degraded releasing materials into the cytosol for cell energy (4). This formation is regulated by different autophagy-related proteins (Atgs) in a highly controlled manner. The Atg8/MAP1LC3 (microtubule-associated protein 1 light chain 3, hereafter referred to as LC3) is necessary for the maturation of the autophagosome (5). LC3 (light chain protein) is a cytosolic protein present in mammalian cells that is converted to LC3-II and recruited to the autophagosome membranes during the autophagic process. It is considered the principal marker of the complete autophagosome and is a reliable protein for monitoring autophagy (6).

The establishment of autophagy is also dependent on another protein, beclin-1, which promotes the recruitment of membranes to form the autophagosome (7). Together with LC3, beclin-1 is also necessary for the occurrence of autophagy (4). The activation of autophagy is represented by an increase in the concentration of these protein pathways (6).

The mammalian target of rapamycin (mTOR) complex is a serine/threonine-protein kinase that forms the central core of two distinct complexes, mTORC1 and mTORC2, which share the ability of autophagy inhibition, but may regulate separate molecular pathways that result in different cellular responses. mTORC1 has been described as a major negative regulator of endosomal biogenesis and autophagy, while the role of mTORC2 in autophagy regulation has been less studied and may act in a different and perhaps complementary manner to that of mTORC1 (8). Several studies report that activated mTOR and impaired autophagy play pivotal roles in inflammatory responses and oxidative stress injury (9,10).

During pregnancy, the regulation of autophagy involves three aspects: the embryo, the mother, and the immune crosstalk at the maternal-fetal interface (2). Some studies reveal that autophagy is present in extravillous trophoblast cells and is important in early pregnancy to promote trophoblastic invasion and uterine vascular remodeling, resulting in normal placentation (11,12). However, the abnormal placentation that occurs in preeclampsia (PE) seems to be the primary cause of placental disorders under perfusion/hypoxia/ischemia, leading to autophagy downregulation and release of factors into the maternal circulation, which generate oxidative stress, inflammatory response, anti-angiogenic factors production, as well as systemic inflammatory response and endothelial dysfunction (12,13).

Preeclampsia is primarily characterized by the onset of clinical parameters such as hypertension and proteinuria from 20 weeks of gestation or by hypertension associated with maternal neurologic or hematologic complications, kidney failure, liver involvement, or fetal growth restriction (14,15). This syndrome affects between 2 to 8% of human pregnancies and constitutes the major cause of maternal and perinatal morbidity and mortality (16). Moreover, PE is classified as early-onset and late-onset PE according to clinical manifestation before or after 34 weeks of gestation, respectively (17). Early-onset PE arises due to defective placentation whereas late-onset PE may be related to the maternal genetic predisposition to cardiovascular and metabolic diseases (18).

Currently, autophagy is being extensively studied in PE, however, the results of the increase or decrease of this process are still controversial. Therefore, the present study sought to investigate the levels of proteins related to autophagy (LC3, beclin-1, and mTOR) in placental tissue from preeclamptic women.

## Material and Methods

### Study population and ethics statement

Placentas were collected from 40 women with singleton pregnancies who were delivered at the Obstetric Unit of Botucatu Medical School, Brazil, by elective cesarean section in all groups. Twenty placentas were collected from normotensive (NT) healthy pregnant women (controls) without hypertension-related complications that delivered at term (≥37 weeks of gestation) and 20 placentas were collected from pregnant women with PE. Gestational age was determined by the date of the last menstrual period and confirmed by early ultrasound examination (<12 weeks of gestation). PE, as defined by the American College of Obstetricians and Gynecologists (17), is the onset of persistently elevated blood pressure (≥140/90 mmHg) with or without proteinuria (≥300 mg in urine collected over 24 h) and with other severe clinical complications after 20 weeks of gestation. Proteinuria was measured in 24-h urine by the Technicon RAXT automation system (Miles Inc., USA), which is a colorimetric method utilized in the Clinical Laboratory of Botucatu Medical School, Brazil. Exclusion criteria included patients in labor, premature rupture of membranes, illicit drug use, and preexisting medical conditions such as diabetes, chronic hypertension, and renal disease.

The study was approved by the Ethics Committee of the Botucatu Medical School, and written informed consent was obtained from all women involved in the study (CAAE number: 17748313.2.0000.5411). All experiments were performed following relevant guidelines and regulations and the Code of Ethics of the World Medical Association (Declaration of Helsinki).

### Sample collection and preparation

All placentas from PE and NT pregnant women were delivered by cesarean section, without labor, and were examined macroscopically according to previous guidelines (19). The samples were processed immediately after delivery, and the tissues were obtained by cutting a cross-section through the full thickness of the placenta including the fetal and maternal surfaces. Tissues with calcification or clots were avoided. Samples of approximately 2 g of placental tissue were used for quantitative real-time polymerase chain reaction (qPCR) and immunohistochemical analysis.

### Expression of transcripts related to autophagy

Placentas obtained from pregnant women with PE and NT were submitted to analysis of the protein-encoding genes *LC3-II*, *beclin-1*, and *mTOR*. Total RNA was extracted from the placentas with the Total RNA Purification Kit (NorgenBiotek Corp., Canada) according to the manufacturer's protocol. After extraction, 1 µg of total RNA was incubated with DNase I Amp Grade (Invitrogen, ThermoFisher Scientific, USA), and measured by Qubit^®^ Fluorometric Quantitation (ThermoFisher Scientific). Subsequently, complementary DNA (cDNA) was synthesized using the ImProm-II^TM^ Reverse Transcription System (Promega, USA) according to the manufacturer's protocol. Quantitative real-time PCR (qPCR) was performed using RT GoTaq^®^ qPCR Master Mix (Promega) according to Matias et al. (20). A 7500 Fast Real-Time PCR System (Applied Biosystems - ThermoFisher Scientific, USA) was used for the analysis. For normalization, GAPDH was used. The primers used in the experiments are listed in Table 1. The differential expression calculation of selected genes was carried out by the data processing method compared with a standard curve (21). To analyze relative gene expression, RNA expression levels in all samples were standardized on a single RNA sample, which was set to a value of 100.

**Table 1:** Primers for autophagy genes and GAPDH.

Target	Gene	Forward primer	Reverse primer	Gene Bank
MAP1LC3	*LC3*	(517) CCAGGAAACCTTCGGCTTCT (536)	(632) CGGTAGAGGCAGCTCAGTTC (613)	NM_032514.3
BECN1	*beclin-1*	(28) TCGCTGAAGACAGAGCGATG (37)	(151) CGATGCTCTTCACCTCGGG (133)	NM_003766.3
MTOR	*mTOR*	(4170) TCGCTGAAGTCACACAGACC (4189)	(4307) CTTTGGCATATGCTCGGCAC (4288)	NM_004958.3
GAPDH	*GAPDH*	(684) CGTGGAAGGACTCATGACCA (703)	(801) GGCAGGGATGATGTTCTGGA (782)	NM_002046.4

Numbers indicate nucleotide positions in the corresponding transcripts.

### Immunohistochemical analysis of placental tissues

Placental fragments were embedded in paraffin, and sections of 4-µm thick slices were cut and placed on histologic slides pretreated with Vectabond (Vector Laboratories Inc., USA). The procedures of deparaffinization, rehydration, and antigen recovery of the material and immunohistochemical analysis of placental tissues from PE and NT pregnant women were performed as described previously (22). Sections of placental tissue were incubated for 60 min at 37°C with antigen-specific primary anti-human antibodies: rabbit polyclonal anti-LC3-II [1/300], anti-beclin-1 [1/100], and anti-mTOR [1/100] (Novus Biologicals, USA) diluted in Antibody Diluent (Cell Marque Co., USA). The expression of autophagy proteins was identified in five random fields of each placental section using an optical microscope (Olympus CX-31, Japan) with 10, 20, and 40× objectives, and a 10× ocular lens. Every section of placental tissue was photographed with a 20× objective and was analyzed with the software ImageJ (NIH, USA). The quantification of the protein analyzed was obtained in pixels/µm/area.

### Statistical analysis

Data from the clinical and laboratory characteristics of pregnant women were analyzed by the non-parametric Mann-Whitney U test. The correlation coefficient (r) was determined using Spearman's correlation coefficient. The statistical program GraphPad Prism version 6.01 (GraphPad, USA) was used, and the statistical significance was set at P<0.05.

## Results

### Subject characteristics

The analysis of clinical and laboratory characteristics of the PE and NT pregnant women is shown in Table 2. There was no statistical difference between the groups regarding maternal age. However, the gestational age at delivery was significantly lower in women with PE than in the NT group. As expected, the PE group showed systolic and diastolic blood pressure and proteinuria values significantly higher, whereas placental weight was significantly lower compared to the NT pregnant women.

**Table 2:** Clinical and laboratory characteristics of pregnant women with preeclampsia (PE) and normotensive (NT) pregnant women.

Characteristics	NT pregnant women(n=20)	Women with PE(n=20)
Maternal age (years)	27 (18-36)	28 (18-41)
Gestational age at delivery (weeks)	38 (37-40)	31* (26-39)
Systolic blood pressure (mmHg)	115 (103-120)	165* (140-210)
Diastolic blood pressure (mmHg)	70 (65-80)	112* (98-120)
Proteinuria (mg/24 h)	<300	3890* (440-19,750)
Placental weight (g)	517 (410-607)	286* (197-515)

Data are reported as median, with the minimum and maximum values in parentheses. *P<0.05 *vs* NT pregnant women (Mann-Whitney U test).

Preeclamptic women involved in the study exhibited severe forms of the disease. Sixty-five percent of these patients were classified as early-onset PE, with gestational age <34 weeks, and 35% were classified as late-onset PE, with gestational age ≥34 weeks, according to Huppertz (17). Proteinuria ≥2 g in 24-h urine collection was detected in 55%, blood pressure ≥160×110 mmHg in 70%, signals of imminent eclampsia in 45%, and eclampsia in 15% of these women. Interestingly, 35% of patients developed an association between two or more of these signals of disease severity.

### Expression of autophagy-related genes

**Figure 1:**
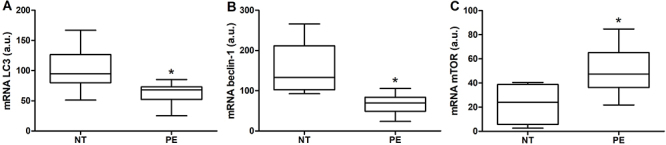
Gene expression of LC3 (**A**), beclin-1 (**B**), and mTOR (**C**) in placental tissues from 20 normotensive pregnant women (NT) and 20 pregnant women with preeclampsia (PE). Results are reported as median (horizontal line), 25th and 75th percentiles (box) and range (whiskers). *P<0.05 *vs* NT (Mann-Whitney U test). a.u.: arbitrary units.

Figure 1A and B shows, respectively, lower gene expression of *LC3* and *beclin-1* in placental tissue from pregnant women with PE compared to the NT group. On the other hand, gene expression of *mTOR* (Figure 1C) was significantly higher in placental tissue from PE women than in the NT group.

### Immunohistochemical analysis of LC3-II, beclin-1, and mTOR

**Figure 2:**
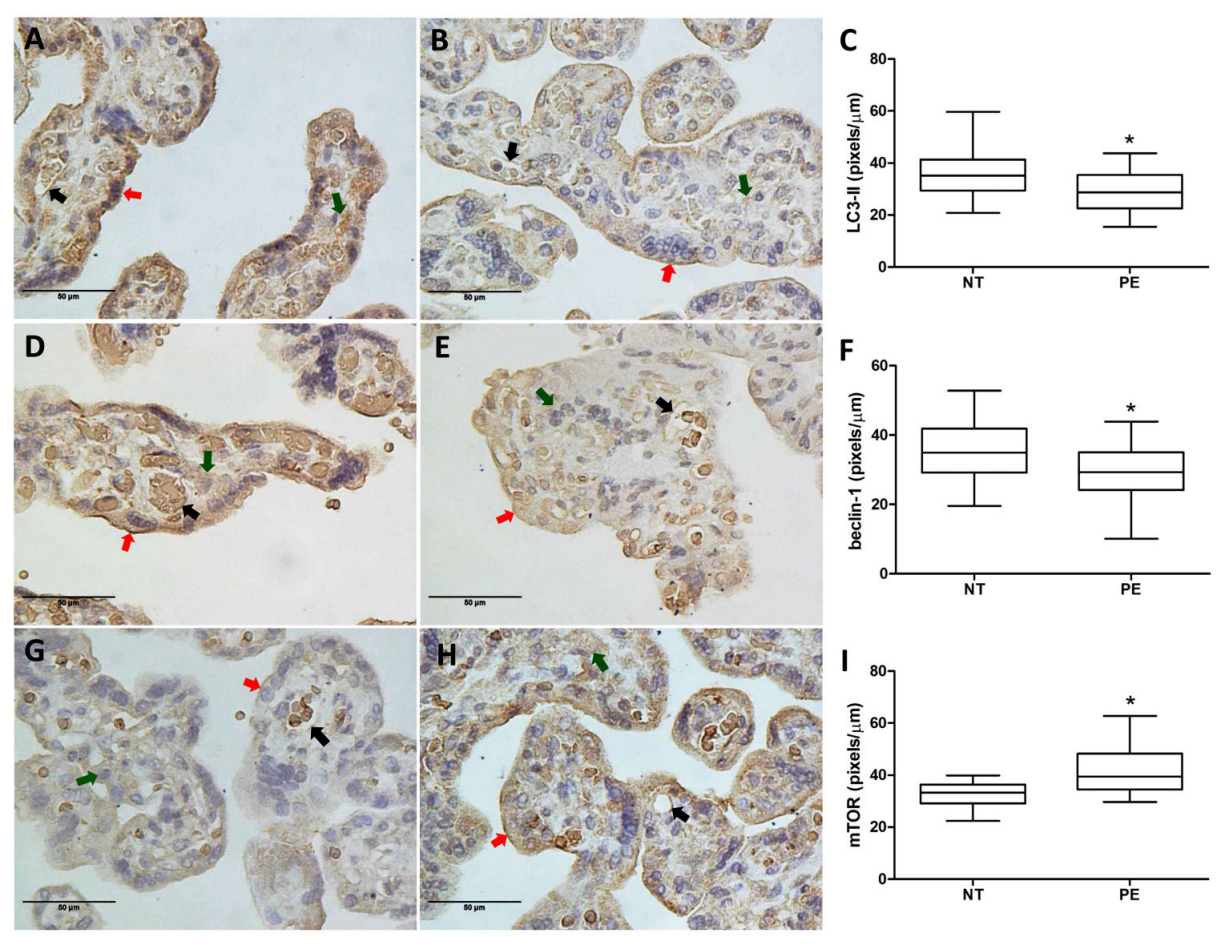
Immunohistochemical staining and quantitative analysis of LC3-II (**A**, **B**, and **C**), beclin-1 (**D**, **E**, and **F**), and mTOR (**G**, **H**, and **I**) in the placenta from 20 normotensive pregnant women (NT) and 20 pregnant women with preeclampsia (PE). The red arrows show positivity in the syncytiotrophoblast, the green arrows show positivity in the cytoplasm of mesenchymal cells, and the black arrows show positivity in the fetal capillary endothelium. Results are reported in pixels/µm and as median (horizontal line), 25th and 75th percentiles (box), and range (whiskers). *P<0.05 *vs* NT (Mann-Whitney U test).

The proteins LC3-II, beclin-1, and mTOR were expressed by syncytiotrophoblast cells (red arrow), mesenchymal cells (green arrow), and endothelial cells of the fetal capillaries (black arrow) as shown in Figure 2. All evaluated proteins showed the same localization in the two groups studied. However, differences in expression intensity were found and quantified using the software Image J. The intensity of LC3-II (Figure 2A, B, and C) and beclin-1 (Figure 2D, E, and F) expression was significantly lower in the placentas of PE women than in the NT group, while mTOR expression was significantly higher in the pregnant women with PE compared to NT pregnant women (Figure 2G, H, and I).

Association analysis between mTOR and LC3-II showed a negative correlation in PE women (r=-0.3433; P=0.0022). No significant correlation between these parameters (r=-0.1905; P=0.1142) was observed in the NT pregnant group. Also, there was no significant correlation between mTOR and beclin-1 in PE (r=-0.2050; P=0.0819) and in NT (r=-0.1612; P=0.1825) groups.

## Discussion

The results of the present study showed a down-regulation in the autophagy proteins in the placenta of pregnant women with PE. Studies of autophagy related to pregnancies complicated by PE are somewhat controversial in the literature (2) and were first reported by Oh et al. (23) followed by others (11,24). Analyses of proteins involved in the initial stage of autophagy activation showed increased LC3-II expression in the placenta from preeclamptic women compared with control pregnant women, while beclin-1 showed no difference between the groups (23). On the other hand, LC3-II overexpression was only observed in the placenta of preeclamptic women when associated with fetal growth restriction (11), and significantly increased expression of LC3 and beclin-1 was detected in placentas from pregnancies complicated by early-onset preeclampsia (25). Akaishi et al. (24) showed by electron microscopy widespread autophagy vacuoles in the syncytial layer and endothelial cells and an increase in LC3-II in patients with PE. Also, autophagy activation, indicated by the increase of LC3-II and decrease of p62 in preeclamptic women, was detected even in the absence of intrauterine growth restriction (24). On the other hand, Goldman-Wohl et al. (26) showed no significant differences in autophagy-associated gene expression in preeclamptic and normal placental samples.

Nakashima et al. (27) showed that the impaired autophagy in the placenta of pregnant women with PE may be a result of a defective lysosomal biogenesis mechanism. According to these results, Ribeiro et al. (28) demonstrated a higher expression of p62 protein in the placentas of preeclamptic women suggesting that there is a reduction in autophagy activity. Together, these reports show that the occurrence of autophagy in the placenta of preeclamptic women deserves further studies.

Regarding mTOR, our results were in line with those of other authors showing that hyperactivation of protein expression in the placenta may be suggestive of an adaptive response to limited nutrient availability or to situations such as hypoxia, which occurs in the placenta of pregnant women with PE, and fetal growth restriction (29).

Another explanation for the discrepancy between our results and the results from the literature (11,23-25) could be the high percentage of preeclamptic women with severe forms of PE in the present study since 65% of the patients were classified as early-onset PE. It has been recognized that this form of PE is associated with higher placental involvement, with more severe lesions, and with an imbalance in cytokines and angiogenic factors, represented by higher TNF-α/IL-10 and sFlt-1/PlGF ratios compared with late-onset PE (30).

In the present study, the high protein expression of mTOR in the placenta of preeclamptic women may be associated with autophagy deficiency. Our results showed a negative correlation between expression of LC3-II and expression of mTOR in the placenta of preeclamptic women suggesting a compensatory regulatory mechanism of autophagy played by mTOR activation in the insufficient and inflamed placenta. mTOR also acts as a central regulator of immune responses by its involvement in differential regulation of pro- and anti-inflammatory cytokine levels (31). Indeed, mTOR can upregulate pro-inflammatory immune responses through the NF-κB (31,32). Thus, NF-kB is considered a potent autophagy inhibitor (33) that is hyperactivated in mononuclear cells of women with PE and associated with higher TNF-α and IL-1β production by these cells (34). NF-kB is activated in response to reactive oxygen species (ROS) and increased hydrogen peroxide production (35). Thus, the high inflammatory and anti-angiogenic state, as well as the elevated NF-κB activation observed in preeclamptic women, could be involved in placental insufficiency, leading to impaired autophagy pathways.

The autophagy and inflammasome pathways are linked by mutual regulation. We recently reported activation of NLRP3 inflammasome in the placenta from preeclamptic women demonstrated by a significant increase of mRNA for NLRP3, caspase-1, IL-1β, TNF-α, and HMGB1. The impaired autophagy in placental tissue of preeclamptic women demonstrated in the present study might be explained by the NLRP3 inflammasome activation in the placenta in PE, already described by our group (22). Considering these recent results of the mTOR regulatory role in autophagy, the association between mTOR activation and inflammation needs to be better studied in PE.

Autophagy controls inflammation through interactions with innate immune pathways, by removing endogenous inflammasome components, and affects the secretion of immune mediators (36). Thus, autophagy induced by inflammatory signals targets ubiquitinated inflammasomes, thereby limiting IL-1β production through inflammasome destruction (37). On the other hand, depletion of beclin-1 and LC3B in mouse macrophages upregulates caspase-1 activation and secretion of mature IL-1β and IL-18 in response to lipopolysaccharide in an NLRP3 inflammasome-dependent manner (38). The blockage of autophagy of macrophage cells with 3-methyl adenine (3-MA) resulted in the production of mitochondrial ROS (mtROS) and NLRP3-dependent IL-1β secretion in the absence of traditional inflammasome stimulants (39). This suggested that damaged mitochondria not be cleared by autophagy release mtROS, which stimulates NLRP3 inflammasomes leading to mature IL-1β secretion (39,40).

In conclusion, we showed that the placenta of women with PE has autophagy deficiency characterized by lower gene and protein expression of LC3 and beclin-1 and higher mTOR expression. Therefore, this autophagy impairment might be dependent on the excessive inflammatory response, on the higher NF-κB activation, and the elevated mTOR expression seen in the placenta of pregnant women with PE. Studies involving cross-talk between autophagy and inflammatory molecular mechanisms in PE might help to better understand the autophagy down-regulation described in the present study.
